# Cost-Effective Modern Chemical Sensor System for Soil Macronutrient Analysis Applied to Thai Sustainable and Precision Agriculture

**DOI:** 10.3390/plants10081524

**Published:** 2021-07-26

**Authors:** Sutasinee Apichai, Chalermpong Saenjum, Thanawat Pattananandecha, Kitti Phojuang, Siraprapa Wattanakul, Kanokwan Kiwfo, Attachai Jintrawet, Kate Grudpan

**Affiliations:** 1 Department of Pharmaceutical Science, Faculty of Pharmacy, Chiang Mai University, Chiang Mai 50200, Thailand; sutasinee.apichai@gmail.com (S.A.); thanawat.pdecha@gmail.com (T.P.); 2 Center of Excellence for Innovation in Analytical Science and Technology (I-ANALY-S-T), Chiang Mai University, Chiang Mai 50200, Thailand; phojuang.kitti@gmail.com (K.P.); siraprapa.wk@gmail.com (S.W.); k.kanokwan11@gmail.com (K.K.); attachai.j@cmu.ac.th (A.J.); 3 Cluster of Excellence on Biodiversity-Based Economics and Society (B.BES-CMU), Chiang Mai University, Chiang Mai 50200, Thailand; 4 College of Arts, Media and Technology, Chiang Mai University, Chiang Mai 50200, Thailand; 5 Department of Chemistry, Faculty of Sciences, Chiang Mai University, Chiang Mai 50200, Thailand; 6 Department of Plant and Soil Sciences, Faculty of Agriculture, Chiang Mai University, Chiang Mai 50200, Thailand

**Keywords:** precision and sustainable agriculture, cost-effective chemical analysis, soil macronutrients, modern chemical sensor system (M-CSS)

## Abstract

A modern chemical sensor system (M-CSS) was developed for the cost-effective chemical analysis of Thai precision and sustainable agriculture (TPSA), which is suitable in rural Thailand and elsewhere. The aim of this study was to achieve precision and sustainable agriculture (P-SA). The M-CSS functions according to the International Union of Pure and Applied Chemistry (IUPAC) definition and incorporates information and communication technologies (ICTs). The developed chemical sensor in the M-CSS is based on a colorimetric determination by a smart device/smartphone. Additionally, the preparation of soil samples was investigated. Soil samples of optimal conditions were extracted using an acid extractant in the ratio of one to two (extract to soil sample). Then, phosphate-phosphorous and potassium were detected with the M-CSS, which showed an excellent correlation with the standard reference methods. Interestingly, it is noteworthy that the at-site analysis of the developed method could detect a greater nitrate-nitrogen content than that of the standard reference method. The developed cost-effective analysis for the plant macronutrient content in the soil, including nitrate-nitrogen, phosphate-phosphorous, and potassium, was demonstrated for organic vegetable farms at the real P-SA research site in Northern Thailand. The obtained results can guide the management of the application of fertilizers. The proposed M-CSS exhibited the potential to be used for at-site soil macronutrient analysis and represents the starting point of Thai precision and sustainable agriculture (TPSA).

## 1. Introduction

The 17 sustainable development goals were adopted by the United Nations in 2015 as part of the 2030 Agenda for Sustainable Development, setting out a 15-year plan to achieve these goals [1]. Among these, Goal 2 (zero hunger), Goal 3 (good health and well-being), Goal 8 (decent work and economic growth), Goal 12 (responsible consumption and production), and Goal 15 (life on land) regard global improvement and have been adopted in Thai precision and sustainable agriculture as well as in relation to reducing inequality [2].

Precision agriculture (PA) is an emerging trend in modern agriculture that has increasingly attracted global interest. A key concept of PA is to “apply the right treatment in the right place at the right time” [3]. PA may be referred to as smart farming or smart agriculture. It presents one of the key ways in which we can achieve the abovementioned sustainable development goals. In recent years, information and communication technologies (ICTs) have also been incorporated into PA practice to increase efficiency in sensing and monitoring.

Recently, developments in PA have been of great interest to researchers from various fields. According to the Scopus database (accessed on 25 May 2021), the number of studies on “precision agriculture” has been dramatically increasing since 1995. However, among the 7170 publications on PA, the number of PA research papers focusing on the subject area of chemistry is small, with only 560 publications to date. Among these, major contributions were from Europe (40%) and Asia (34%), followed by North America (14%), South America (8%), Africa (2%), and Australia (2%). Interestingly, among all chemical research on PA, there have been less than 20 previously reported analytical chemistry studies. It was found that only 26 articles involved soil-related analysis, 14 articles focused on nutrients, and only 13 articles were related to soil nutrients.

One challenge in the development of chemical analysis for real-world application is the general trends of research focusing on high-performance instrumental methods, which have limited the applicability, in real applications, of the subject at hand. On the other hand, although demand for simple indicator-type sensors (without extra power required and that anyone can use) is high, the interest gained for research development of such sensors is considerably low [4].

In this study, attempts were made to develop a modern chemical sensor system (M-CSS) as a cost-effective chemical analysis tool that can be applied to PA with the goal of producing alternative PA that is appropriate for sustainable agriculture in rural areas, including parts of Thailand and elsewhere.

**Figure 1:**
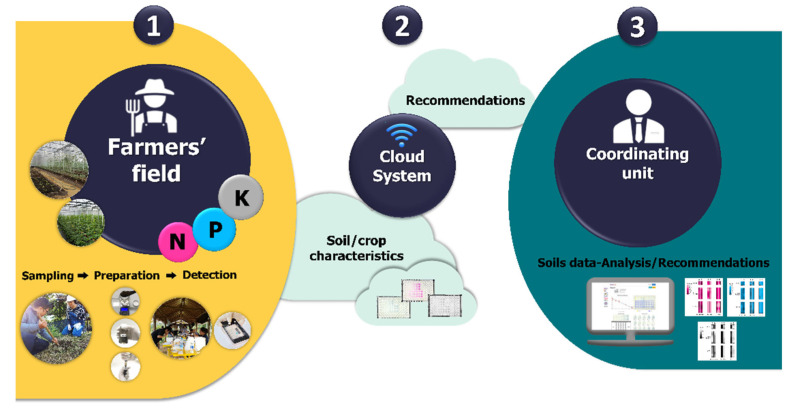
The three main components of modern chemical sensor systems for Thai precision and sustainable agriculture (M-CSS-TPSA). The 1, 2, and 3 components are the on-site chemical analysis, a cloud system of ICTs, and the coordinating unit staffed by experts.

The M-CSS consists of three main components, as illustrated in Figure 1. The first component is the on-site chemical analysis. It was designed for simple operations that could be carried out by inexperienced people, for example, a farmer or one of the farmers’ family members, even those of school age. The second component involves a cloud system of ICTs, which engages data management for data access from any place at any time. The last component refers to the center or coordinating unit staffed by a pool of experts with various expertise (such as chemical analysis, soil sciences, plant sciences, etc.). By employing the M-CSS, samples could be taken and analyzed at sites with the aid of photography in color using a mobile phone or smartphone involving chemical sensors. The transformed signals could be sent to the cloud system, and image processing would be carried out to provide the results of the nutrients contained in the soil. The experts would make use of the information to provide suggestions for the farmers.

Cost-effective chemical sensors for nutrients, including nitrate-nitrogen (nitrate-N), phosphate-phosphorous (phosphate-P), and potassium, were developed for Thai precision and sustainable agriculture (TPSA) in this study. According to the International Union of Pure and Applied Chemistry (IUPAC) [5], a chemical sensor is defined as a device that transforms chemical information, ranging from the concentration of a specific sample component to total composition analysis, into an analytically useful signal. It consists of two basic functional units: a receptor part and a transducer part [6]. This proposed chemical sensor is based on a colorimetric determination that occurs from the chemical reaction. The specific chemical reactions for nitrate-nitrogen (nitrate-N), phosphate-phosphorous (phosphate-P), and potassium include diazotized-coupling of the N-(1-naphthyl)-ethylenediamine (NED) reaction, molybdenum blue reaction, and Kalibor turbidimetric reaction, respectively. The reagent concentration and incubation time are optimized, and the procedure is designed for ease of use. A smart device/smartphone camera is used to detect the color that occurs. Free software is used to transduce signals that are proportional to such macronutrient contents. The accuracy and precision of the developed chemical sensor are excellent when compared with those of the reference methods. Moreover, the preparation of soil samples is investigated, namely, the type of extractant and the ratio of extraction. The optimal sample preparation can be achieved using the acidic extractant at one to two ratios of extract to soil samples. Finally, the developed cost-effective modern chemical sensor system is successfully demonstrated on-site in northern Thailand for organic vegetable farms.

## 2. Results and Discussion

### 2.1. Performance of Proposed Chemical Analysis

The chemical sensors in the M-CSS were developed based on colorimetric methods. The procedures were developed as described in Section 3.3. Each well plate was designed for simultaneous determination with two duplicate standard sets (2 × 5 wells), with the other 50 wells for samples. It should be noted that the outer wells were not in use due to the light effects that occur with photography. The proposed nitrate-N assay involved the reactions of nitrate to nitrite and the coupling of a nitrite ion with NED to produce a pink product [7,8]. The mixed solution of nitrate-N standard and reagent A (see Section 3.2) was incubated for 15 minutes before adding reagent B. The mixture was then incubated for one minute after adding reagent B (see Section 3.2) for color detection. The assays of phosphate-P and potassium had fewer steps than that of nitrate-N because the color reaction could be produced by a reagent. The incubation time for the phosphate-P and potassium assays was one minute. After the incubation period, the colors were detected with a smart device camera and transformed into a signal value to create a calibration curve and evaluate the concentration of analyte (nitrate-N, phosphate-P, or potassium).

**Figure 2:**
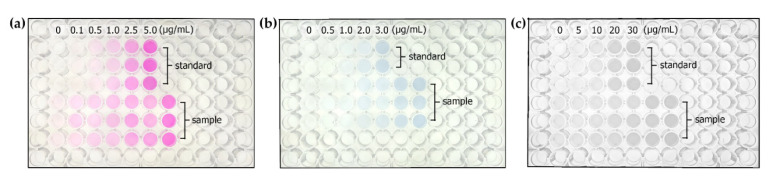
Examples of photos obtained from M-CSS-TPSA: (**a**) nitrate-N, (**b**) phosphate-P, and (**c**) potassium.

Calibration graphs were created to plot the intensity of color and the concentration of an analyte, as presented in Figure S1a–c. Figure 2a–c present the resulting colors forming in a well plate for nitrate-N (with a linear calibration: G intensity = 20.94 [nitrate-N] + 0.941, R^2^ = 0.997), phosphate-P (with a calibration: R intensity = 15.67 [phosphate-P] + 0.312, R^2^ = 0.997), and potassium (with a calibration: B intensity = 1.017 [potassium] + 0.9079, R^2^ = 0.996), respectively. The linear ranges were 0-5.0 μg/mL, 0–3.0 μg/mL, and 0-30.0 μg/mL for nitrate-N, phosphate-P, and potassium, respectively. The analytical performance was evaluated through performance parameters including accuracy and precision under repeatability conditions. The obtained percentages for recoveries and relative standard deviations (RSDs) are summarized in Table 1. The recovery was calculated as the ratio of the expected concentration to the proposed concentration. The results showed moderate recovery in the range of 80–115, while the percentages of RSD were less than 11. This was in accordance with the standard format and guidance for the Association of Official Analytical Chemists (AOAC) Standard Method [9].

### 2.2. Soil Extraction

#### 2.2.1. Effect of Soil Extraction Solutions on Colorimetric Reactions

**Figure 3:**
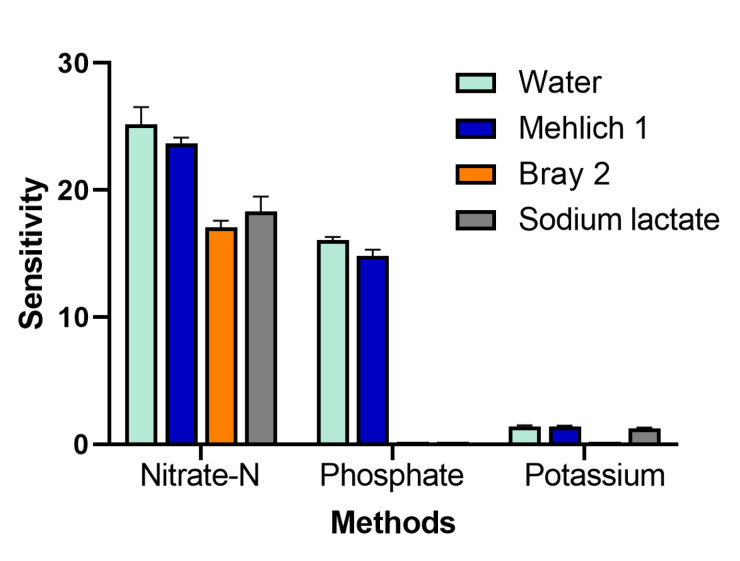
Effect of soil extraction solutions on sensitivity for the determination of nitrate-N, phosphate-P, and potassium (n = 3).

The effect of the extraction solutions on the colorimetric reactions investigated indicated the sensitivity of the method, which is the differential between the color intensity and concentration unit. The cadmium reduction and Griess reaction employing diazotized-coupling of NED for the determination of nitrate-N were differently affected by each extraction solution. The pink color of the product deepened in the water or acidic extractant (so-called Mehlich 1) and faded in Bray 2 and sodium lactate extractants. The results showed that the sensitivities of nitrate-N determination in water and Mehlich 1 extractant were higher than in Bray 2 and sodium lactate extractants. For determination of phosphate-P by molybdenum, a blue reaction presented no effect from the water or the Mehlich 1 extractant. On the other hand, the Bray 2 and sodium lactate extractants affected this reaction greatly. The molybdenum blue reaction was not present in the Bray 2 and sodium lactate extractants. However, there was an effect on the Kalibor turbidimetric reaction for the determination of potassium. Overall, the high sensitivities were present in water and the Mehlich 1 extractant, whereas the Bray 2 and sodium lactate extractants were the cause of the reduction in the sensitivities, as shown in Figure 3. The results showed excellent reproducibility in triplicate.

#### 2.2.2. Extraction Efficiency of Soil Samples

**Figure 4:**
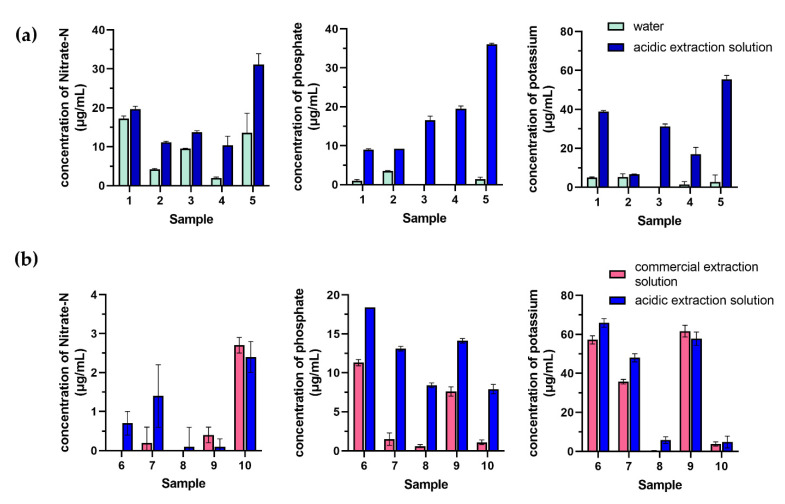
Extraction efficiency of soil samples by their acidic extraction, comparing (**a**) water and (**b**) the commercialized soil extraction for determination of nitrate-N, phosphate-P, and potassium. Error bars are standard deviations from triplicates.

The extraction efficiencies of soil samples with water, the acidic extractant, and the commercialized soil extractant were also investigated. Five soil samples were extracted for the determination of nitrate-N, phosphate-P, and potassium. The results showed that the obtained nitrate-N, phosphate-P, and potassium concentrations by acidic extraction were significantly higher than those obtained by water extraction, as presented in Figure 4a. The extraction efficiency of phosphate-P by the acidic extractant was significantly higher than that by the commercialized soil extractant. There was no significant difference between the acidic extractant and the commercialized soil extractant for the extraction of nitrate-N and potassium, as shown in Figure 4b. The statistical analysis assessing these comparisons was the paired t-test at a confidence level of 95%.

#### 2.2.3. Effect of the Extraction Ratio on Extraction Efficiency

**Figure 5:**
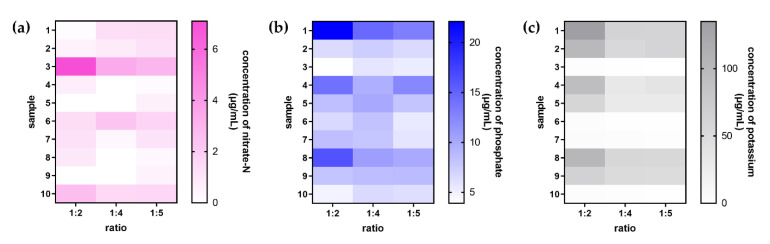
Extraction efficiency of soil samples according to the acidic extraction of different soils, showing the extraction solution ratio for the determination of (**a**) nitrate-N, (**b**) phosphate-P, and (**c**) potassium.

The nitrate-N, phosphate-P, and potassium ions were extracted from ten soil samples by different ratios of soil to the acidic extractant to investigate extraction efficiency. The paired t-test at a confidence level of 95% was applied to a statistical analysis assessing the three ratios. The results found that the extraction efficiencies of nitrate-N and phosphate-P ions had no statistically significant differences. However, the 1:2 ratio showed better extraction efficiency of potassium ions than of others. Figure 5 is a heat map showing the average concentration of each ion that was obtained in triplicate.

### 2.3. Method Correlation

**Figure 6:**
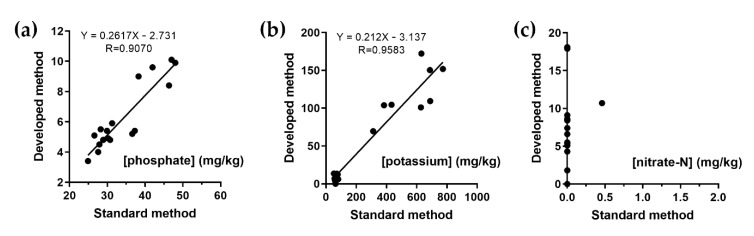
Correlation of determination of (**a**) phosphate-P, (**b**) potassium, and (**c**) nitrate-N by the reference method and the proposed method.

Twenty collected soil samples were extracted and analyzed by the standard reference methods of soil analysis compared with the proposed method. Highly significant correlations at the 95% level were obtained for the phosphate-P and potassium determinations with the statistical correlation coefficients of 0.9070 and 0.9583, respectively, as shown in Figure 6a,b. As shown in Table 2, the ratio correlations between the standard method and the proposed phosphate and potassium methods were 6 ± 1 and 5 ± 1, respectively. These differences may result from the different soil sample preparations that the units of reference method reported in the mg of nutrient per kg of dry soil. Another reason may be the different conditions of extraction, including the type of extractant and time. Unfortunately, there was no relation between both methods for the determination of nitrate-N. Most of the soil samples that were analyzed by the standard reference method were not found to have nitrate ions, as shown in Figure 6c. This may be the result of a change in nitrogen form or disappearance during the preservation and shipping of soil samples to the laboratory. For this reason, the M-CSS has evolved as a model system to overcome such problems, carrying out at-site analysis. However, only one type of soil sample was demonstrated. The effect of soil type was further investigated, but this is not further explored here. The soil samples will be classified into 12 types according to the soil texture triangle based on the U.S. Department of Agriculture (USDA) particle size classification [10], and the correlation of the type with the nutrient contents in the soil obtained by the standard and the proposed methods will be examined.

### 2.4. Application to Real Agriculture

At the demonstrated organic vegetable farm, the positions of the sampling sites were registered using a GPS navigator device. The soil samples and cultivations were obtained by photographs using a smartphone camera, such as those shown in Figures S2–S4. The advantages gained from the above include traceability, in which the time and location of the analysis operations can be traced back. Moreover, such information can help experts to decide how to carry out effective planning. The macronutrient contents in the soils were monitored in the stages of soil preparation, cultivation, and harvest, and then the fertilized and unfertilized plots were compared. The macronutrient contents in the soils were obtained via the proposed method and were used for mapping, as shown in Figures S5–S7. Evidently, the cultivation stage showed a decrease in nitrate-N within the soil in fertilized plots; however, there was a higher nitrate-N content in unfertilized plots. This evidence indicates that the nitrate type of nitrogen is crucial in the early growth stages and agrees with various field investigations utilizing N-labeled fertilizer that have shown that nitrogen uptake is primarily derived from the soil rather than a fertilizer [11]. However, though increased nitrogen fertilization of the crop may result in high nitrogen uptake, it is not always used to increase biomass production. Moreover, excessive nitrogen fertilizer use reduces nitrogen use efficiency, raises production costs, and pollutes the environment [12,13]. The results showed that the trends of the phosphate-P and potassium contents were different from that of the nitrate-N content. Increases in phosphate-P and potassium contents were found in fertilized plots, but decreases were observed in unfertilized plots. In fertilized plots, it was observed that plants took phosphorus from supplementary inputs because of the slow phosphorus release rates from the soil; simultaneously, over-application of phosphorus caused accumulation in the soil [14,15]. The analysis results demonstrated that spatial data may be useful for research into the management of fertilizer application and organic farming, which is an element of precision farming. The operation could be cost-effectively carried out by farmers in rural areas by networking with experienced contacts in towns via an Internet connection.

## 3. Materials and Methods

### 3.1. Chemicals and Reagents

All chemicals used were of analytical grade; acetic acid, ammonium fluoride, hydrochloric acid, phosphoric acid, sodium hydroxide, and sulfuric acid were obtained from Merck, Germany. N-(1-naphthyl)-ethylenediamine dihydrochloride was obtained from Sigma-Aldrich. L-lactic acid was obtained from TCI, Japan. Sulfanilamide was obtained from Fluka, Japan. NitraVer^®^6 reagent, PhosVer^®^3 reagent, and Potassium 3 reagent Permachem^®^ Powder were obtained from HACH, USA, and were manufactured in 2020.

### 3.2. Chemical Preparation

#### 3.2.1. Reagents

Reagent A for the nitrate assay was prepared by dissolving a NitraVer^®^6 reagent pack in 5 mL deionized (DI) water. To prepare reagent B, 10 mL of 85% phosphoric acid and 1 g of sulfanilamide were dissolved in 80 mL water. After dissolving completely, 0.1 g of N-(1-naphthyl)-ethylenediamine dihydrochloride was added and adjusted to 100 mL with DI water. Four PhosVer^®^3 reagent packs were dissolved in 5 mL DI water as the reagent of the phosphate assay. The reagent of the potassium assay was prepared by dissolving a Potassium 3 reagent pack in 5 mL DI water.

#### 3.2.2. Soil Extraction Solutions

The acidic extractant (so-called Mehlich 1) is composed of 0.05 M hydrochloric acid and 0.0125 M sulfuric acid. To prepare the Bray 2 extractant, 1.11 g of ammonium fluoride was dissolved in 0.1 M hydrochloride 1 L. A mixture of 0.11 M lactic acid, 0.4 M acetic acid, and 0.1 M sodium hydroxide was prepared to produce a sodium lactate extractant.

### 3.3. Proposed Chemical Analysis Procedure

The analyses were based on color-forming reactions that utilize the same principles as the standard or reference methods: diazotized-coupling of NED [8], the molybdenum blue method [16], and turbidimetry with tetraphenylborate [17] for nitrate-N, phosphate-P, and potassium, respectively. Apart from conventional laboratory-prepared reagents, several types of ready-to-use chemicals and reagents were employed for convenience of use in the field (at-site analysis). These include the products obtained from HACH, USA.

**Figure 7:**
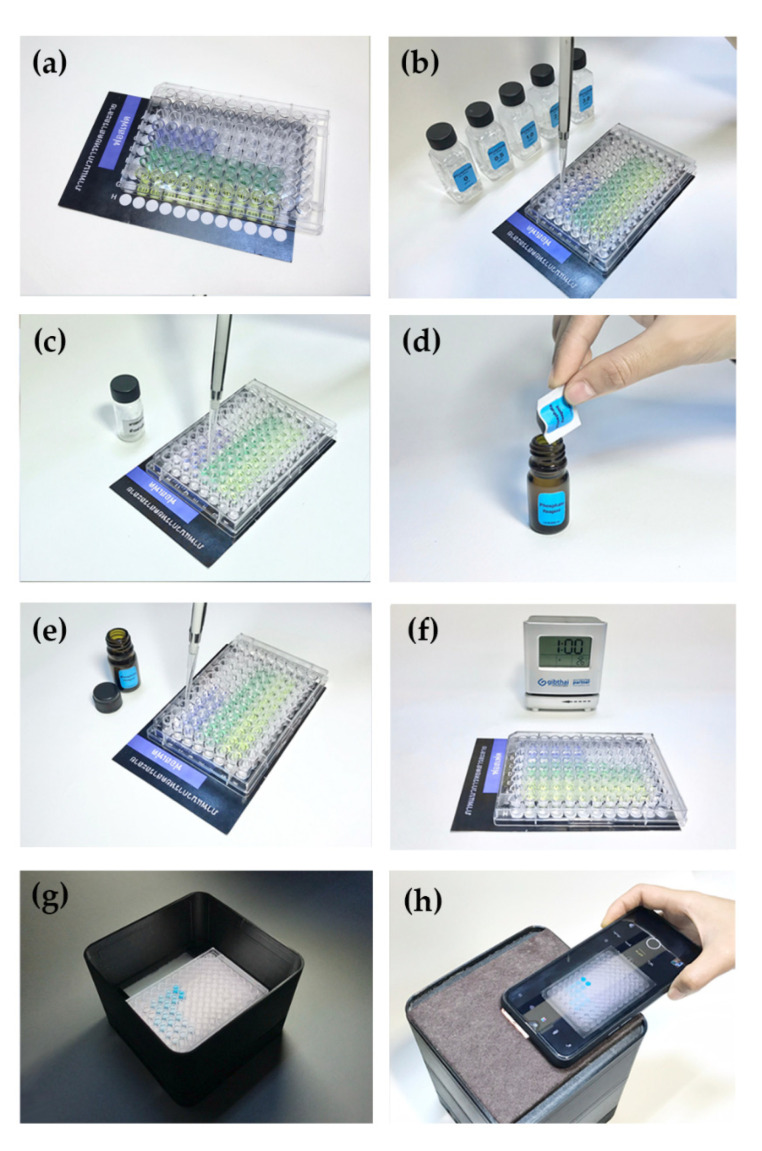
Simple procedure of modern chemical sensor system for Thai precision and sustainable agriculture (M-CSS-TPSA). Figures were (**a**) placing microplate on a pad, (**b**) adding standard solution, (**c**) adding sample, (**d**) preparing a reagent, (**e**) mixing the sample with reagent, (**f**) incubation, (**g**) light-controlled box, and (**h**) detection.

Instead of the conventional milliliter volume operation, the chemical analysis was downscaled with microliter volumes using a micropipette, a microplate with 96 wells (Corning, USA), and a smartphone as a detector. Accordingly, 200 μL of the sample and 50 μL of reagent solution were fixed for all assays. In detail, the analytical procedure consisted of the following steps (as shown in Figure 7): (a) a microplate was placed on a pad for the guide taking the solution to a micro-well; (b) the standard solution was taken to the well; (c) the sample was placed in the well; (d) the reagent was prepared by dissolving it in ID water; (e) the reagent was mixed with the sample solution; (f) the mixture was incubated before measurement of color; (g) this microplate was placed in a lightbox; (h) the colors were detected by a smart device camera (Apple iPhone8, China) on a light-control box [18].

Evaluation of the concentration of the analyte was made via image processing with free software, such as Image J software [19], or with our laboratory-developed web service, that is, the modern chemical sensor system by Thailand Science Research and Innovation (TSRI) and Center of Excellence for Innovation in Analytical Science and Technology (I-ANALY-S-T) where an analyte concentration can be directly read on a smart device. Our laboratory developed the web service on the cloud. On the web service, the photograph taken is uploaded and color values are translated to signals. Such data are saved on the cloud, which is publicly accessible. In the case of fewer skilled persons, after taking a photograph of the well plate with the resultant forming color, the photo can be uploaded via the Internet and the evaluation can be carried out by an individual at the central laboratory, with the evaluated analyte concentration sent to the person at the site (farmer). In principle, the image captured by a smart device’s camera is analyzed to extract the color values that relate to the concentration of the analyte. A calibration graph is plotted to compare the concentration of the standard with the intensity of the color seen, to evaluate the concentration in the sample.

### 3.4. Soil Extraction

#### 3.4.1. Effect of Soil Extraction Solutions on Colorimetric Reactions

The effect of soil extraction solutions on the diazotized coupling of the NED reactio n, molybdenum blue reaction, and Kalibor turbidimetric reaction was studied. Water, Mehlich 1, Bray 2, and the sodium lactate solution, which are extractants of plant nutrients from the soil, were used as the solvent for dissolving the standard of nitrate-N, phosphate-P, and potassium. The colorimetric reactions for the determination of nitrate-N, phosphate-P, and potassium were produced as described in Section 3.3. The colorimetric signals were expressed through the intensity of color and used to create a calibration curve to compare the effects of soil extraction solutions.

#### 3.4.2. Extraction Efficiency of Soil Samples

The extraction efficiency of soil by the acidic extractant was investigated and compared with that of water and a commercial soil extractant. To extract nitrate-N, phosphate-P, and potassium from the soil samples, 2.5 g of soil was weighed and shaken in 5 mL of the different extractants for five minutes. After shaking, the extract was set aside until observations were carried out of the separation between the silt and solution. The liquid layer was filtered through a nylon filter (0.45 µm, ID 25 mm, MACHEREY-NAGLE, Germany). The clear extract solution was measured for the contents of nitrate-N, phosphate-P, and potassium as described in Section 3.3.

#### 3.4.3. Effect of Extraction Ratio on Extraction Efficiency

In total, 5 g of the soil sample was weighed and shaken in the acidic extractant for five minutes to extract nitrate-N, phosphate-P, and potassium. The ratio of the soil sample weight (g) to the extractant volume (mL) was investigated at 1:2, 1:4, and 1:5. After extraction, the emulsion was left to stand for 10 min and filtered through a nylon filter. The clear filtrate was analyzed as described in Section 3.3 to compare the extraction efficiencies of nitrate-N, phosphate-P, and potassium for the soil samples at each ratio.

### 3.5. Method Correlation

In total, 5 g of soil was weighed and shaken in 10 mL of the acid extractant for five minutes. Then, it was left to stand for 10 minutes before filtering with a nylon filter. The filtrate was analyzed for nitrate-N, phosphate-P, and potassium via the proposed method. In the standard method for nitrate, soil samples were extracted by potassium chloride according to ISO 14256 [20]. Ammonium fluoride in the acidic medium and ammonium acetate of pH 7 were used for the extraction of the available phosphorus and potassium ions, respectively [21,22]. The soil extracts were detected via the spectrophotometric method for nitrate and available phosphorus [7,16]. Potassium ions were analyzed by atomic emission spectroscopy (AES).

### 3.6. Real World Application

The potential of the developed cost-effective chemical analyses with information systems was demonstrated for real use in research at an organic vegetable farm, Saraphi District, Chiang Mai, Thailand. The produce was organic vegetables, including green oak, Japanese cucumber, and long bean. Green oak was planted in 0.5 × 1 m plots. The plots of Japanese cucumber and long bean were trenches each of the size 0.5 × 18 m. A comparison of the macronutrient contents of the soil in two plots, namely, a manure treatment plot and a non-fertilizer plot, was carried out. The soil nutrient contents were monitored by the developed method in each growth period of the organic vegetables.

At the planned sampling sites, smartphone and GPS navigator devices were employed to locate the positions of the sampling sites. Samples were taken from a depth of 15 cm from the ground soil and collected in plastic bags. The soil samples were collected, photographed, and labeled with brief information before being analyzed. Three soil samples per plot of green oak and six soil samples per plot of Japanese cucumber and long bean were collected. A sample portion (5 g) in the acidic extraction solution (10 mL) was shaken for 5 min and left to stand for 10 minutes before filtering with a nylon filter. The filtrate was analyzed for nitrate-N, phosphate-P, and potassium.

## 4. Conclusions

Our attempt to develop a modern chemical sensor system (M-CSS) for the cost-effective analysis of plant macronutrient contents in soil was successful. The resulting developed sensors responded to nitrate-N, phosphate-P, and potassium with adequate sensitivity, reproducibility, and a fast response time. This investigation included the preparation of the soil samples from which the selected extraction solution demonstrated excellent efficiency in extracting nitrate-N, phosphate-P, and potassium ions. The M-CSS was applied to real use for research at an organic vegetable farm. The results of a comparison between fertilized and unfertilized plots can guide the management of the application of fertilizers. The developed M-CSS exhibited the potential to be used for at-site soil macronutrient analysis and represents the starting point of Thai precision and sustainable agriculture.

## Figures and Tables

**Table 1:** Percentages of recovery and relative standard deviation (RSD; n = 3).

Assay	Concentration (μg/mL)		Recovery (%)	RSD (%)
	Proposed	Expected		
**Nitrate-N**	0.3 ± 0.0	0.25	101	7.1
	0.7 ± 0.1	0.75	98	9.1
	1.5 ± 0.1	1.50	98	7.1
	2.3 ± 0.1	2.50	91	2.8
	3.6 ± 0.1	3.50	102	4.0
**Phosphate-P**	0.8 ± 0.1	0.75	104	9.9
	1.0 ± 0.1	1.50	96	8.1
	1.9 ± 0.1	2.00	95	4.7
	2.6 ± 0.3	2.50	103	10.7
	3.1 ± 0.3	3.00	105	8.0
**Potassium**	12.9 ± 0.5	12.50	103	3.7
	16.6 ± 1.2	15.00	111	7.3
	20.6 ± 1.9	20.00	103	9.3
	25.3 ± 1.3	25.00	101	5.3
	28.0 ± 0.2	30.00	93	0.6

**Table 2:** Ratio of the obtained nutrient contents, comparing the amounts obtained from the proposed method to those obtained with the reference method.

Sample No.	Phosphate-P Content (mg/kg)			Potassium Content (mg/kg)		
	Proposed	Reference	Ratio	Proposed	Reference	Ratio
**1**	9	38	4	104	384	4
**2**	5	37	7	12	74	6
**3**	5	30	6	152	774	5
**4**	8	46	6	150	688	5
**5**	10	42	4	12	77	6
**6**	5	28	6	109	689	6
**7**	6	31	5	101	628	6
**8**	10	47	5	105	434	4
**9**	3	25	7	14	60	4
**10**	4	28	7	7	56	8
**11**	5	31	6	15	63	4
**12**	10	48	5	172	631	4
**13**	5	37	7	70	312	4
**14**	6	28	5	11	63	6
**15**	5	29	6	13	72	5
**16**	5	30	6	14	52	4
**17**	5	27	5	16	81	5

## Data Availability

The data presented in this study are available on request from the corresponding author.

## Supplementary Materials

The following are available online at https://www.mdpi.com/article/10.3390/plants10081524/s1. Figure S1: Calibration curves for (a) nitrate-N, (b) phosphate-P, and (c) potassium based on color values. Error bars are standard deviations from triplicates. Figure S2: Sampling soil while preparing the soil for plantation. Figure S3: Organic vegetables including (a) green oak, (b) Japanese cucumber, and (c) long bean (at about two weeks of growth). Figure S4: Organic vegetables, including (a) green oak, (b) Japanese cucumber, and (c) long bean, were ready to be harvested at about four to six weeks of growth. Figure S5: Soil nitrate-N contents were obtained via the developed method in each growth period of organic vegetables. Comparison of nitrate-N content in soil between (a) manure treatment and (b) non-fertilizer. Figure S6: Soil phosphate-P contents were obtained via the developed method in each growth period of organic vegetables. Comparison of phosphate content in soil between (a) manure treatment and (b) non-fertilizer. Figure S7: Soil potassium contents were obtained via the developed method in each growth period of organic vegetables. Comparison of potassium content in soil between (a) manure treatment and (b) non-fertilizer.
