# Supplementary material for: Cost-Effective Modern Chemical Sensor System for Soil Macronutrient Analysis Applied to Thai Sustainable and Precision Agriculture
Source: Plants (Basel). 2021 Jul 26;10(8):1524. doi: 10.3390/plants10081524 (PMC8401004; doi:10.3390/plants10081524)
Supplement: Supplementary file 1 [file plants-10-01524-s001.zip › plants-1287319-supplementary.pdf]

Article

Cost-Effective Modern Chemical Sensor System for Soil Macronutrients Analysis Applied to Thai Sustainable and Precision Agriculture

Sutasinee Apichai [1,2,3], Thanawat Pattananandecha [1,2,3], Kitti Phojuang [2], Siraprapa Wattanakul [2,4], Attachai Jintrawet [2,5], Kanokwan Kiwfo [2,3] ,Chalermpong Saenjum [1,2,3,*], and Kate Grudpan [2,3,6]

1 Department of Pharmaceutical Science, Faculty of Pharmacy, Chiang Mai University, Chiang Mai, 50200, Thailand; sutasinee.apichai@gmail.com (S.A.); thanawat.pdecha@gmail.com (T.P.); chalermpong.saenjum@gmail.com
2 Center of Excellence for Innovation in Analytical Science and Technology (I-ANALY-S-T), Chiang Mai University, Chiang Mai, 50200, Thailand; phojuang.kitti@gmail.com (K.P.); siraprapa.wk@gmail.com (S.W.); k.kanokwan11@gmail.com (K.K.); kgrudpan@gmail.com (K.G.)
3 Cluster of Excellence on Biodiversity-Based Economics and Society (B.BES-CMU), Chiang Mai University, Chiang Mai, 50200, Thailand
4 College of Arts, Media and Technology, Chiang Mai University, Chiang Mai, 50200, Thailand
5 Department of Plant Science and Natural Resources, Faculty of Agriculture, Chiang Mai University, Chiang Mai, 50200, Thailand
6 Department of Chemistry, Faculty of Sciences, Chiang Mai University, Chiang Mai, 50200, Thailand
* Correspondence: chalermpong.s@cmu.ac.th; Tel.: +66-89-950-4227

Supplementary Materials

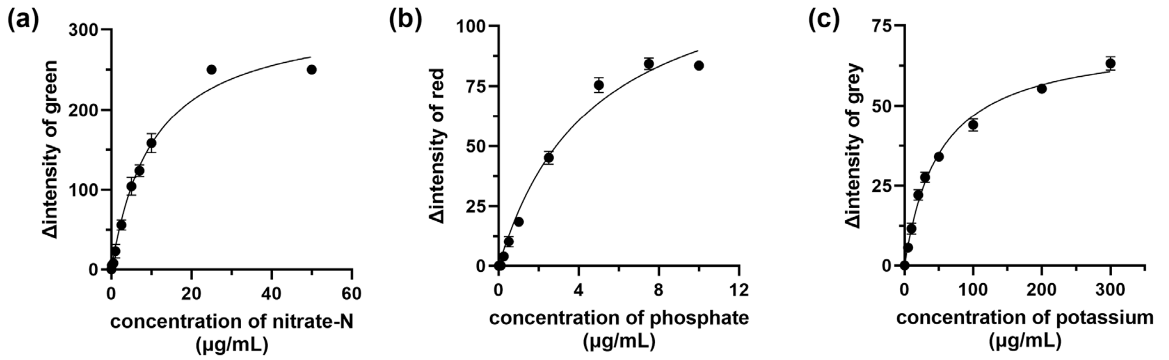

Figure S1 Calibration curves for (a) nitrate-N, (b) phosphate, and (c) potassium based on color values. Error bars are standard deviations from triplicates.

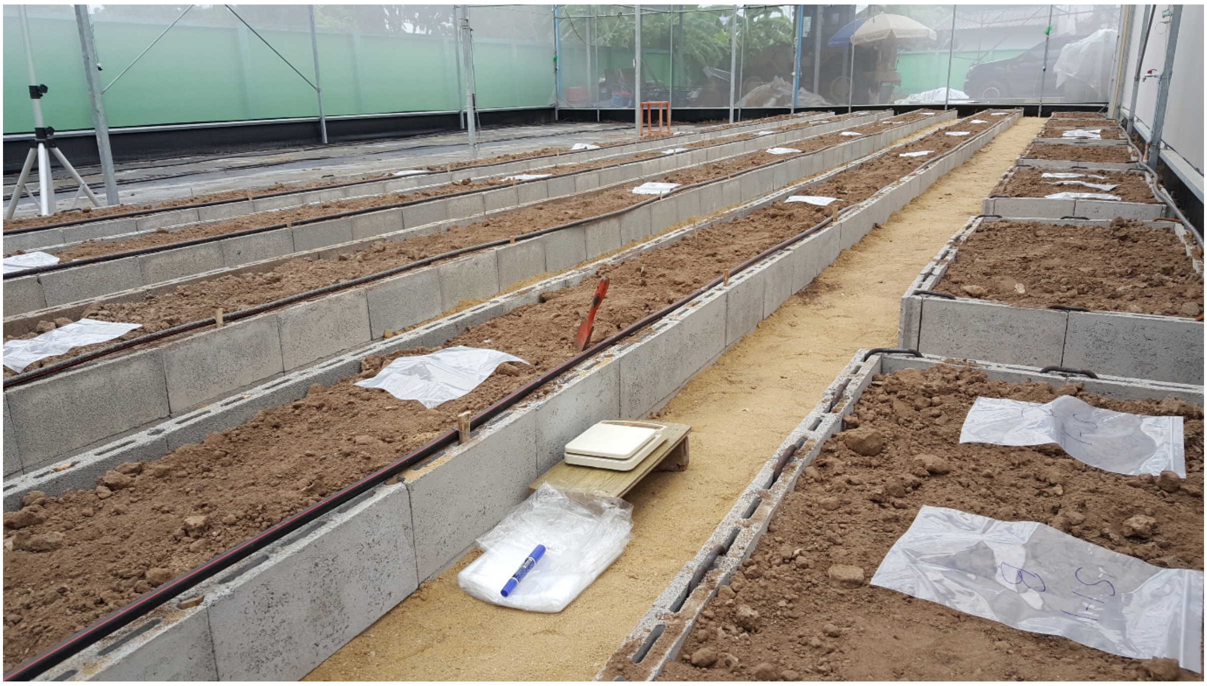

Figure S2 Sampling soil samples while preparing the soil for plantation.

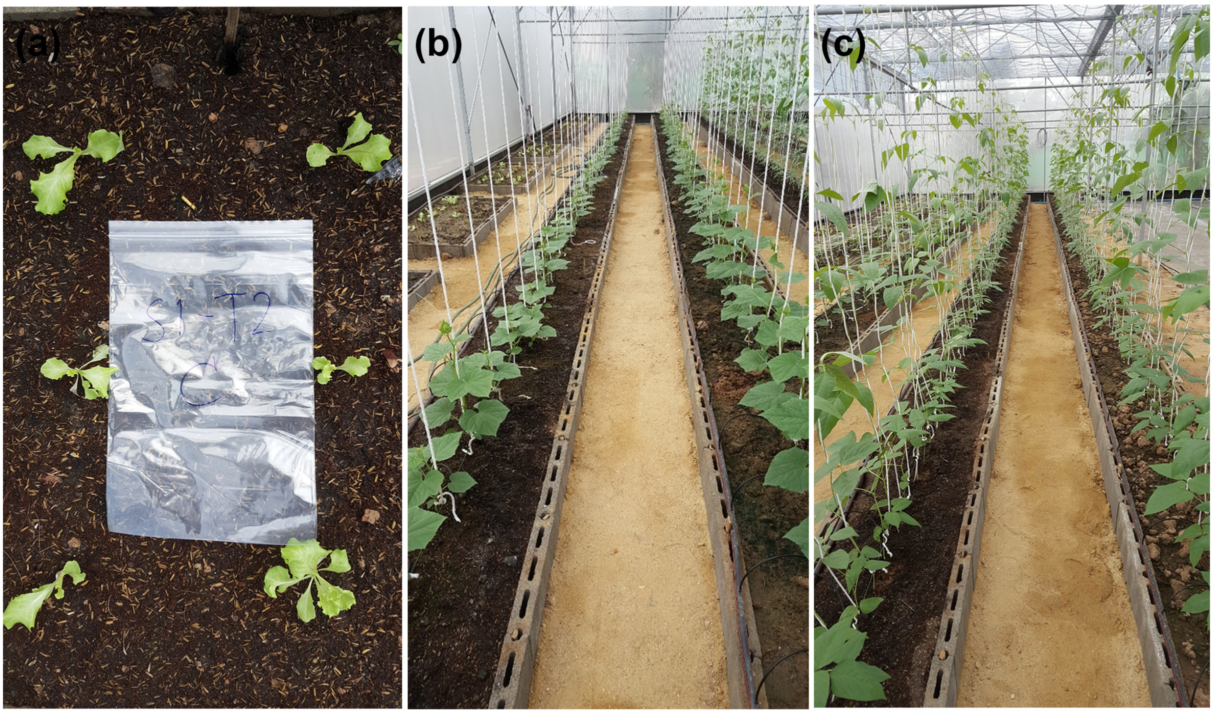

Figure S3 Organic vegetables that include (a) Green Oak, (b) Japanese Cucumber, and (c) Long Bean about two weeks of growth.

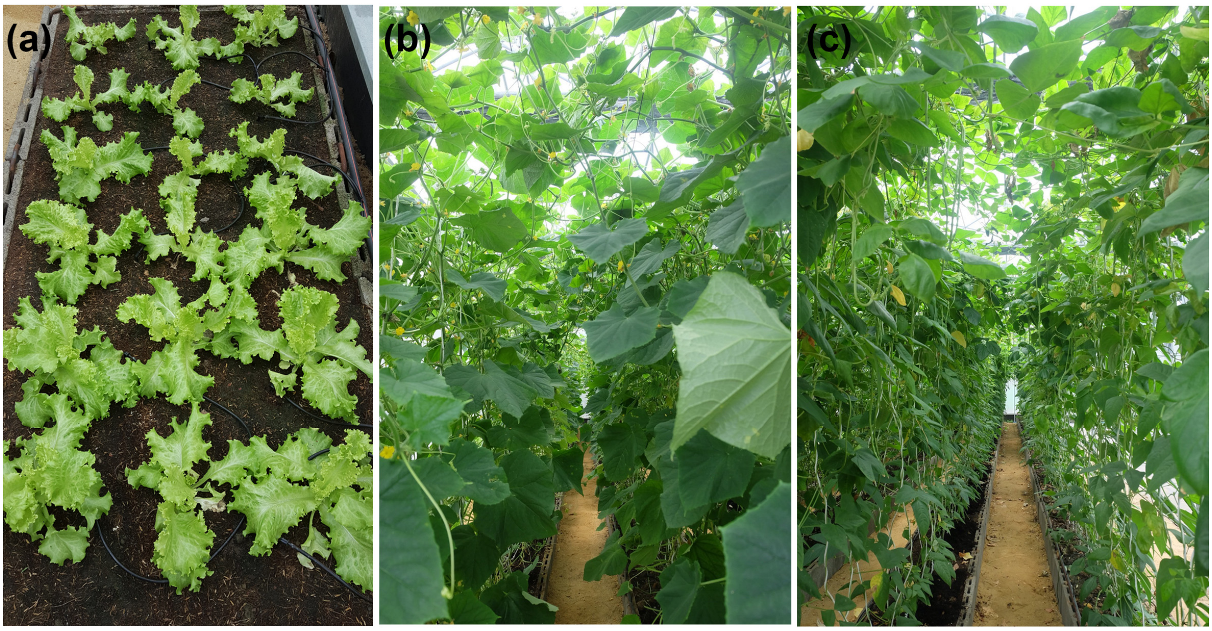

Figure S4 Organic vegetables, which include (a) Green Oak, (b) Japanese Cucumber, and (c) Long Bean, were ready to harvest about four to six weeks of growth.

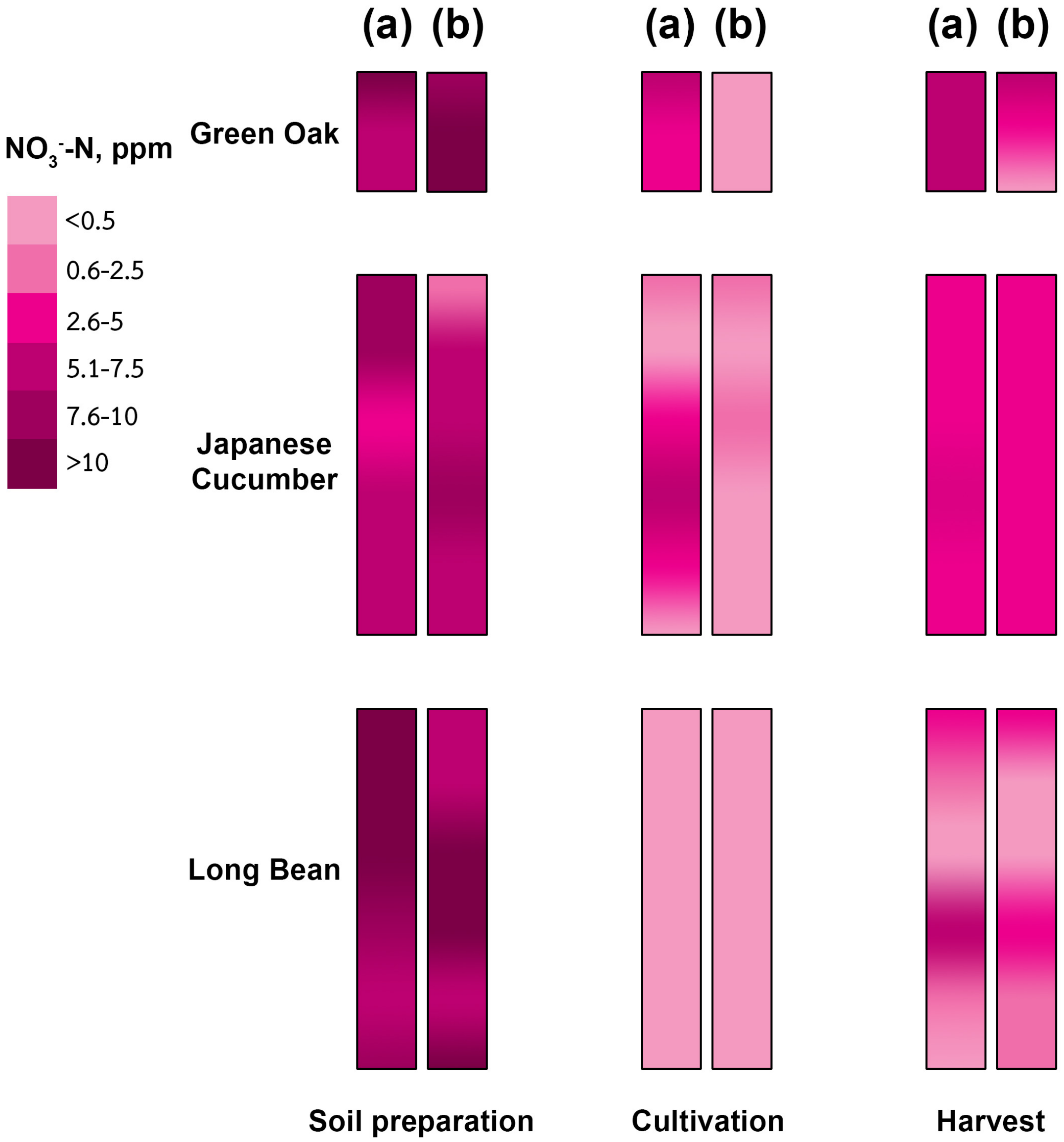

Figure S5 The soil nitrate-N contents were obtained by the developed method in each growth period of organic vegetables. Comparison in nitrate-N content in soil between (a) manure treatment and (b) non-fertilizer.

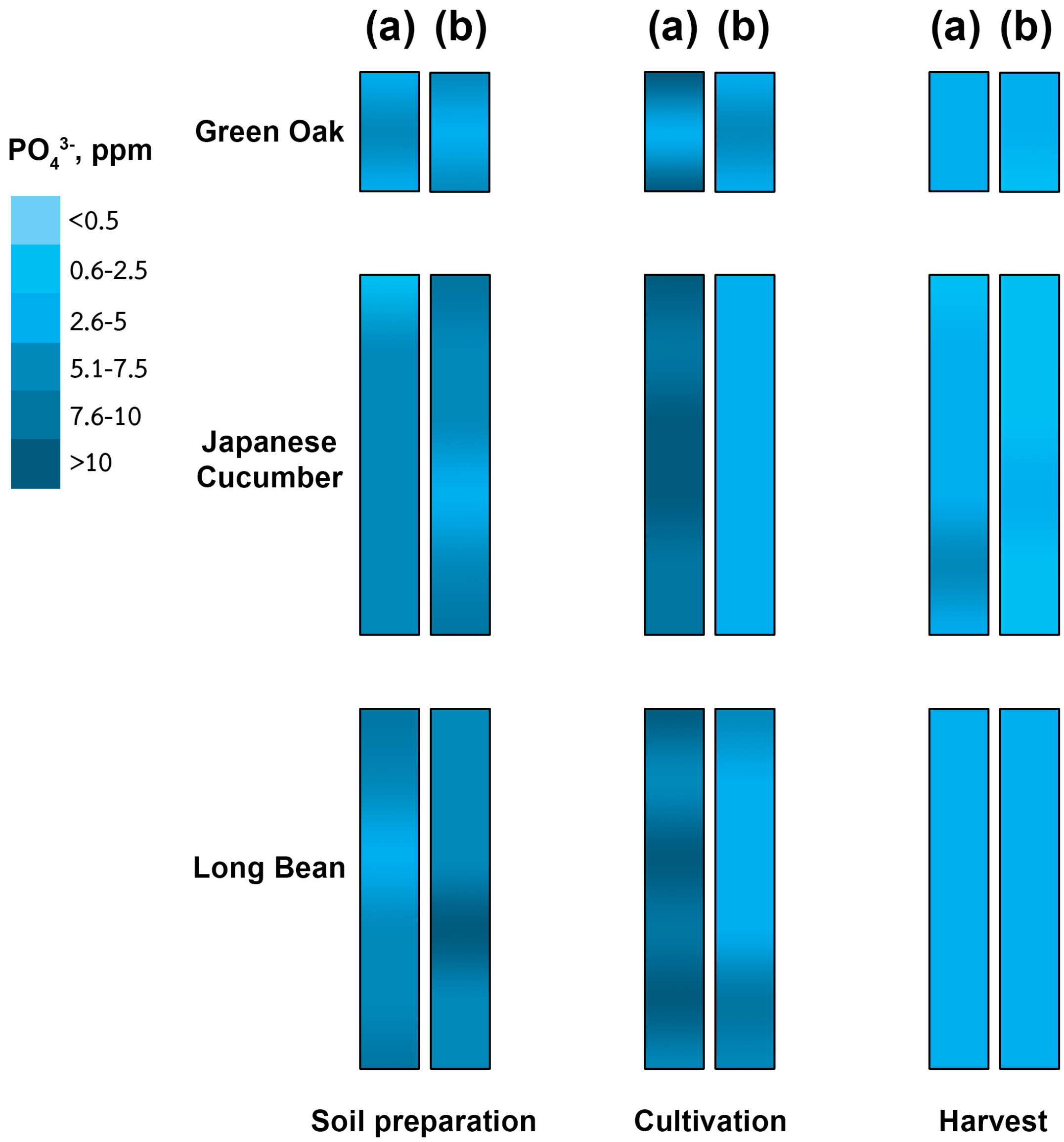

Figure S6 The soil phosphate contents were obtained by the developed method in each growth period of organic vegetables. Comparison in phosphate content in soil between (a) manure treatment and (b) non-fertilizer.

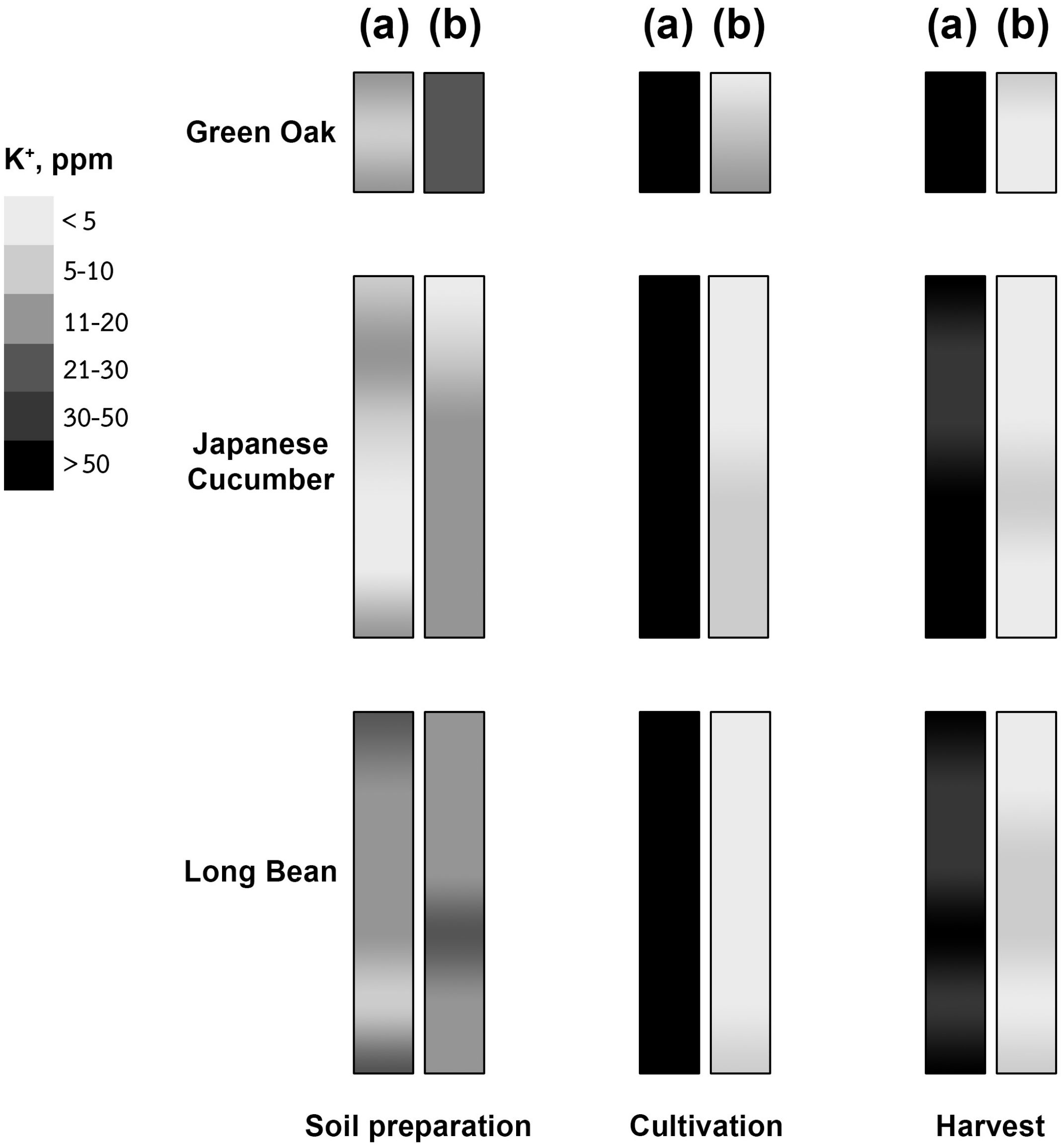

Figure S7 The soil potassium contents were obtained by the developed method in each growth period of organic vegetables. Comparison in potassium content in soil between (a) manure treatment and (b) non-fertilizer.
